# Specific tractography differences in autism compared to developmental coordination disorder

**DOI:** 10.1038/s41598-022-21538-0

**Published:** 2022-11-14

**Authors:** Emily Kilroy, Marzio Gerbella, Lei Cao, Peter Molfese, Christiana Butera, Laura Harrison, Aditya Jayashankar, Giacomo Rizzolatti, Lisa Aziz-Zadeh

**Affiliations:** 1 Brain and Creativity Institute and USC Chan Division of Occupational Science and Occupational Therapy, University of Southern California, 3620A McClintock Avenue, Los Angeles, CA 90089-2921 USA; 2 Department of Medicine and Surgery, University of Parma, 43100 Parma, Italy; 3 Center for the Developing Brain, Child Mind Institute, New York, NY USA; 4 Center for Multimodal Neuroimaging, National Institutes of Mental Health, Bethesda, MD 20892 USA

**Keywords:** Autism spectrum disorders, Developmental disorders

## Abstract

About 85% of children with autism spectrum disorder (ASD) experience comorbid motor impairments, making it unclear whether white matter abnormalities previously found in ASD are related to social communication deficits, the hallmark of ASD, or instead related to comorbid motor impairment. Here we aim to understand specific white matter signatures of ASD beyond those related to comorbid motor impairment by comparing youth (aged 8–18) with ASD (*n* = 22), developmental coordination disorder (DCD; *n* = 16), and typically developing youth (TD; *n* = 22). Diffusion weighted imaging was collected and quantitative anisotropy, radial diffusivity, mean diffusivity, and axial diffusivity were compared between the three groups and correlated with social and motor measures. Compared to DCD and TD groups, diffusivity differences were found in the ASD group in the mid-cingulum longitudinal and u-fibers, the corpus callosum forceps minor/anterior commissure, and the left middle cerebellar peduncle. Compared to the TD group, the ASD group had diffusivity differences in the right inferior frontal occipital/extreme capsule and genu of the corpus callosum. These diffusion differences correlated with emotional deficits and/or autism severity. By contrast, children with DCD showed unique abnormality in the left cortico-spinal and cortico-pontine tracts.

*Trial Registration* All data are available on the National Institute of Mental Health Data Archive: https://nda.nih.gov/edit_collection.html?id=2254.

## Introduction

Autism Spectrum Disorder (ASD) is a neurodevelopmental disorder characterized by a heterogeneous presentation of social communication deficits, repetitive and restrictive behaviors^1^, and sensorimotor deficits^2^. The complexity of ASD makes it difficult to investigate the etiology and identify underlying neural mechanisms of the disorder. Abnormalities in ASD white matter remain unclear. Both hyper- and hypoconnectivity have been demonstrated in children with ASD across multiple tracts. These include a number of motor-related tracts (cortico-spinal tract, cerebellar tracts, cortico-pontine tracts)^3–6^ as well as tracts important for interhemispheric coordination^3–5,7^.

One tract commonly found to be abnormal in ASD is the cingulum^6^. The cingulum is a complicated tract implicated in numerous neurological and psychiatric conditions, including schizophrenia, depression, post-traumatic stress disorder, obsessive compulsive disorder, mild cognitive impairment, and Alzheimer’s disease^8^. The cingulum connects many cortical and subcortical territories with the cingulate cortex, a cortical region involved in many aspects of social, emotional, and motor behavior^9^. In particular, much of the anterior and mid-cingulum, and the neighboring cingulate cortex, appear to be important for regulating motor behavior on the basis of social, interoceptive, and motivational efforts^9,10^. In addition to the cingulum, many other tracts have been implicated in ASD, as compared to typically developing (TD) peers, including the splenium of the corpus callosum and the cerebellar peduncles, which are both also involved in aspects of sensorimotor functioning^4^. Given that these tracts are related to motor functioning, it is possible that white matter abnormalities are related to motor impairments rather than the social deficits that are the hallmark of ASD. Indeed, about 85% of children with ASD present motor difficulties, and either have or are at risk of comorbid developmental coordination disorder (DCD)^2,11^. In fact, studies suggest that individuals with ASD have substantial overlap in motor impairments with DCD, and qualify for DCD on gold standard DCD assessments, such as the Movement Behavioral Assessment for Children (MABC^12–19^; see for a systematic review^20^). Nevertheless, ASD and DCD groups may significantly differ on more social motor tasks, such as imitation (ASD with significantly poorer imitation)^18^. Further, white matter studies on DCD groups have also found diffusivity differences in several motor tracts previously implicated in ASD, including the descending tracts, cerebellar tracts, and the splenium of the corpus callosum^21^. Thus, many of the white matter differences that have been previously reported in ASD could be due to motor comorbidity rather than related to core ASD symptoms. To the best of our knowledge, no research has yet compared white matter tracts between TD, ASD, and DCD participants. Thus here, for the first time, we disentangle motor impairments from social impairments in diffusivity differences in ASD by directly comparing ASD to a matched group of children with DCD, as well as a TD group, and correlate diffusivity findings with both social and motor measures.

## Methods

### Participant characteristics

Participants aged 8 to 17 with ASD *(n* = 30; 4 = female), probable DCD *(n* = 24; 7 = female), and TD controls *(n* = 35; 5 = female) participated in the study (note, ASD is more prevalent in males)^22^. Informed consent was obtained from all participants or a parent/legal guardian for participants under 16 years old. All experimental protocols were approved by the University of Southern California Office for the Protection of Research Subjects and carried out in accordance with the Declaration of Helsinki.

All participants were born after 36 weeks of gestation and screened for MRI compatibility and their capacity to give informed consent. Other inclusion criteria included: (a) full-scale IQ >  = 80 (in cases where the full-scale IQ was less than 80, participants were included if their verbal IQ score or perceptual reasoning IQ score was greater than 79 as assessed by the Wechsler Abbreviated Scale of Intelligence, Second Edition;14 (b) right handed as assessed by a modified Edinburgh questionnaire;15 (c) no history of loss of consciousness greater than five minutes; (c) sufficiently fluent in English and parent with English proficiency. Participants were recruited from clinics in the greater Los Angeles healthcare system, through local public and private schools, and social media advertising. Written consent was acquired in accordance with the study protocols approved by the University of Southern California Institutional Review Board. Three ASD participants and four DCD participants were on psychotropic medication at the time of data collection. ASD participants received a diagnosis either through a clinical ASD diagnostic interview, an ASD diagnostic assessment, or both. Eligible participants with ASD had a previous clinical diagnosis and met the criteria on the Autism Diagnostic Observation Schedule, Second Edition (ADOS-2^23^), the Autism Diagnostic Interview-Revised (ADI-R^24^), or both, which were administered by trained experimenters and overseen by clinical psychologists at the time of the study.

Additional inclusion criteria for the ASD group and the probable DCD group included no diagnosis of other neurological or psychological disorders except for attention deficit hyperactivity disorder (ADHD) or generalized anxiety disorder. Further, inclusion criteria for the DCD group also included a score less than 15% on the Movement Assessment Battery for Children, Second Edition (MABC-2)^16^ and a score indicating a likely presence of a DCD diagnosis on the Developmental Coordination Disorder Questionnaire (DCDQ)^25^. DCD and TD participants were excluded if they had: (a) a diagnosis or an immediate family member with a diagnosis of ASD, or (b) a Social Responsiveness Scale-2 (SRS-2) T-score indicating risk of ASD (> 60) combined with an ADOS-2 score in the clinical range. TD controls were also excluded if they had any neurological or psychological disorder including ADHD and generalized anxiety disorder or if they scored below the twenty-fifth percentile on the MABC-2 score or were suspected to have a DCD diagnosis based on their DCDQ score.

### Behavioral and psychological measures (see Supplementary Material for specific details)

Two behavioral motor measures were administered to capture a wide range of motor skills in ASD and DCD, the MABC-2 and the Florida Apraxia Battery^17,18^ modified for children (FAB-M)^26^. The MABC-2^27^ was used as a performance-based assessment to evaluate motor skills. The MABC-2 is one of the most widely used tools to measure motor skills in school-aged children and the only assessment currently widely recommended to measure clinically relevant motor deficits^28^. It is comprised of three subsections: Manual Dexterity, Balance, and Aiming and Catching. Praxis skills were assessed using the FAB-M^26,29,30^, which consists of Gesture to Command (GTC), Meaningful Imitation (GTI MF), Meaningless Imitation (GTI ML), and Tool Use (TU; gestures using a physical tool). The Social Responsiveness Scale, 2nd Edition (SRS-2)^31^, a parent survey, was used to assess social deficits. Alexithymia was measured using the 20-item self-report Alexithymia Questionnaire for Children (AQC)^32^. The ADOS-2^23^ and the ADI-R^24^ were conducted and used to assess autism, with the ADI-R, the Reciprocal Social Interactions (RSI) subscore used to index autism social severity. The Sensory Over-Responsivity (SenSOR) parent-report inventory was used to assess sensory sensitivities^33^. The Repetitive Behavior Scale-Revised (RBS-R) parent-report questionnaire was used to measure repetitive behaviors^34,35^. To measure symptoms of attention deficit hyperactivity disorder (ADHD), the child self-report measure of the Conners 3rd edition ADHD Index^36^ (CCR) was used.

### Behavioral analysis

To compare groups in social and motor skills a one-way analysis of variance (ANOVA) test was conducted for each behavioral variable and all results *p* < 0.05 are reported (Table 1).

**Table 1:** Group Demographics.

	Controls *N* = 21			ASD *N* = 22			DCD = 16		
	Mean	SD	Range	Mean	SD	Range	Mean	SD	Range
Sex (sum)	8 F			6 F			6 F		
Age	11.98	2.24	8.8–17.8	11.41	2.06	8.6–17.4	11.79	2.27	8.7–15.3
Full-Scale IQ	111.00	8.19	98–125	113.73	20.37	72–156	110.25	17.78	74–135
AQC	6.81	4.24	0–14.0	8.67	5.22	0–20	7.5	4.57	2.0–18
CCR^a,b^	6.90	5.15	0–21.0	12.27	6.91	1.0–24	11.31	6.70	0–23
SRS Total^a,b,c^	45.95	4.93	39–55	77.45	2.37	53–90	55.81	8.24	42–69
RBS Total^a,c^	45.75	7.83	43–71	67.86	16.41	52–108	49.31	4.88	43–60
SenSOR Total^a,b,c^	3.66	3.58	0–14	24.5	12.23	2–45	10.43	10.50	1–37
MABC-2 Total^a,b,c^	10.57	1.66	8–14	5.76	2.37	1–10	4.31	1.85	1–7
GTC^a,b^	0.714	0.13	0.44-0.92	0.55	0.166	0.24-0.88	0.611	0.17	0.32-0.84
IMI MF^a,b,c^	0.74	0.10	0.48–96	0.44	0.185	0.16-0.84	0.54	0.18	0.36-0.92
IMI ML^a,b^	0.61	0.18	0.33-0.1	0.426	0.197	0.11-0.78	0.39	0.12	0.22-0.56
TU^a,b^	0.83	0.082	0.65-0.94	0.57	0.16	0.29-0.82	0.61	0.19	0.24-0.94
ADOS-2	–	–	–	6.22	2.02	1–9	–	–	–
ADI-R RSI	–	–	–	19.25	5.82	12–29	–	–	–

Table of sample demographics. a = TD > ASD; b = TD > DCD; c = ASD > DCD at p < .05; AQC = Alexithymia Questionnaire for Children 2-factor Total; SRS Total = Social Responsivity Scale Total; RBS Total = Repetitive Behaviors Scale Total; SenSOR Total = Sensory Over-Responsivity Scale Total; MABC-2 Total = Movement Assessment Battery for Children-2 Total Scorel; CCR = Connors Child Report; GTC = gesture to command; IMI MF = imitation of meaningful gestures; IMI ML = imitation of meaningless gestures; TU = tool use; ADOS-2 = Autism Diagnostic Observational Scale-2 Comparison Score; ADI-R RSI: Autism Diagnostic Interview Revised, Reciprocal Social Interactions Scale.

### MRI data acquisition, processing, and analysis

For data collection, motion analysis, and pre-processing, see Supplementary Materials.

#### Cingulate U-fibers Region of Interest (ROI)

We used DSIstudio to reconstruct the cingulate U-fibers and subdivide them according to the parcellation of Hau and colleagues^5^. For each ROI (left and right hemisphere: caudal anterior, isthmus, posterior, and rostral anterior cingulate U-fibers; Table 3), tractography parameters included: tracking threshold set to random; angle = 55°–65°; step size = 0.5; random smoothing; PRIMARY seed orientations, default otzu = 0.6. The tracking algorithm was stopped after 50,000 seeds.

#### Tractography Analysis: DSI Studios

*Group differences:* Whole Brain: Diffusion MRI correlational tractography^37^ was used to study the effect of group status on quantitative anisotropy (QA), mean diffusivity (MD), axial diffusivity (AD), and radial diffusivity (RD). A multiple regression model was used to consider group status, age, full-scale IQ, and sex separately for each group contrast (TD:ASD, TD:DCD, ASD:DCD). A T-score threshold of 2.5 was assigned to select local connectomes, and the local connectomes were tracked using a deterministic fiber tracking algorithm^37^. An FDR threshold of 0.05 was used to select tracts. To estimate the false discovery rate, a total of 4000 randomized permutations were applied to the group label to obtain the null distribution of the tract length.

#### Cingulum U-Fiber ROIs

Linear regression was performed using group as the dependent variable and three covariates (age, sex, and full-scale IQ) for each diffusivity (QA, MD, RD, AD) in each ROI.

#### Behavior correlations

In tracts with significant between group differences, diffusivity metrics (QA, MD, RD, AD) were correlated via Pearson’s partial correlation coefficients with the following behavioral data: AQC 2-factor total [see Supplementary Materials for 2-factor calculation], CCR Total, SRS-2 Total, RBS Total, SenSOR Total, MABC-2 Total, the GTC, IMI MF, IMI ML, TU sections of the FAB-M, ADOS-2 Comparision Score (ASD only), ADIR RSI (ASD only). To limit the number of comparisons, we only investigated behavioral measures that we hypothesized would be related to each tract based on previous research (e.g., motor tracts). We further restricted our analysis by only correlating measures in the clinical group(s) that significantly differed from the TD group (e.g., if the tract was significantly different between the TD and ASD groups we only looked at a-priori correlations in the ASD group). A-priori hypothesized significant correlations (*p* < 0.05), and trends (*p* < 0.06) are reported.

## Results

### Behavioral results

Group demographics, characteristics, and significant differences are reported in Table 1. According to the MABC-2, 80% of the ASD participants scored in the motor difficulty range. Age, sex, and full-scale IQ did not significantly differ between groups (*p* < 0.05). Additional statistical information regarding group differences can be found in Supplementary Materials (Table 1S).

### QA tractography contrasts

**Figure 1:**
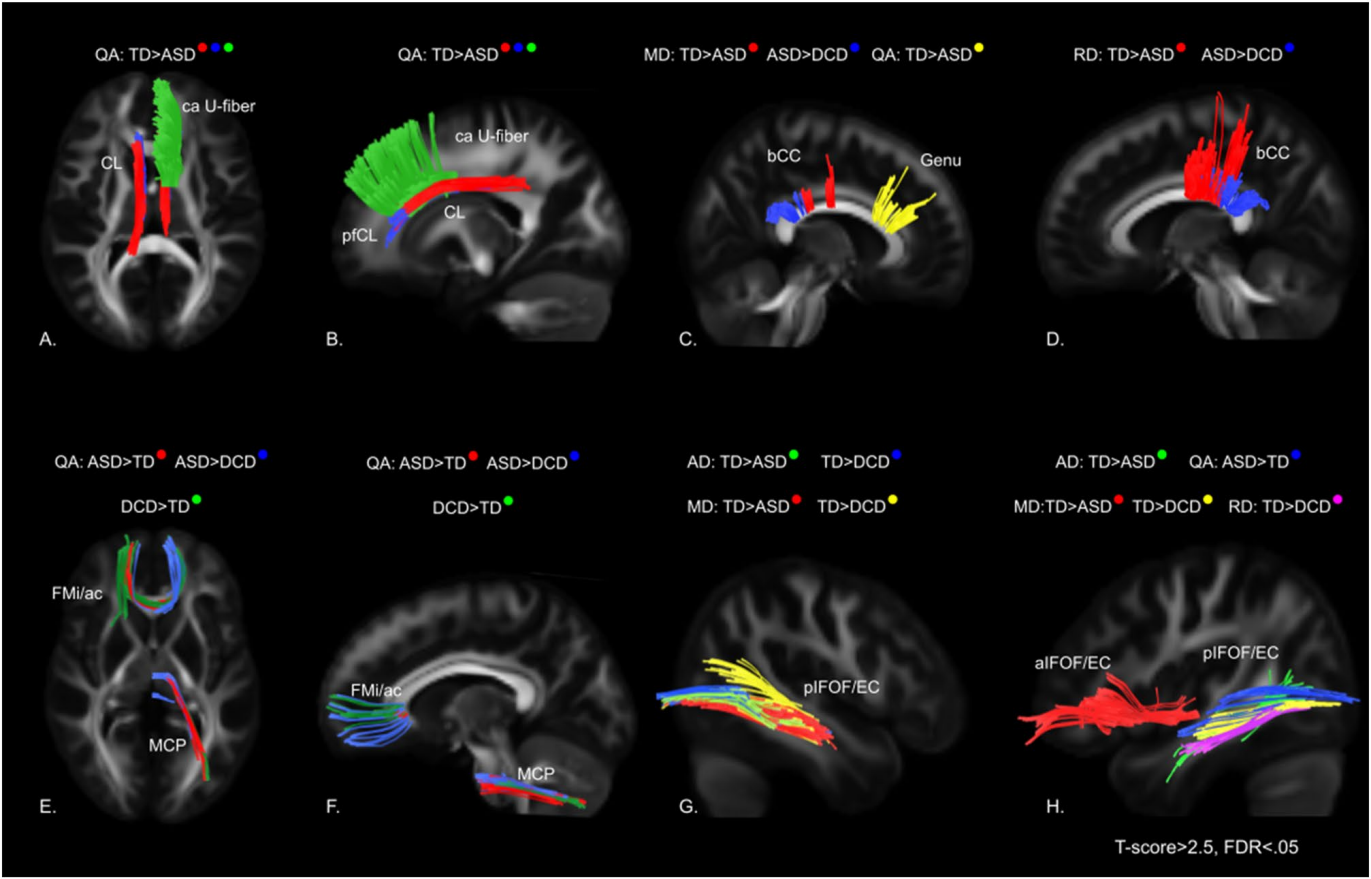
Select tracts from whole brain group contrast analysis. (**A**,**B**) QA: bilateral fronto-parietal cingulum (TD > ASD = red), bilateral parolfactory cingulum (TD > ASD = blue), right caudal anterior u-fiber (TD > ASD = green). (**C**) MD: body of the corpus callosum (ASD > DCD = blue; TD > ASD = red), QA: genu (TD > ASD = yellow). (**D**) RD: Corpus Callosum (TD > ASD = red, ASD > DCD = blue). (**E**,**F**) QA: Forceps minor/anterior commissure (ASD > DCD = blue, ASD > TD = Red, DCD > TD = Green), QA left middle cerebellar peduncle (ASD > DCD = blue, ASD > TD = Red, DCD > TD = Green). (**G**) Left posterior IFOF/EC: AD TD > DCD (Blue); AD TD > ASD (Green); MD TD > ASD (Red); MD TD > DCD (Yellow); H. Right posterior IFOF/EC: AD TD > ASD (Green); MD TD > ASD (Magenta); MD TD > DCD (Yellow); QA ASD > TD (Blue); Right anterior IFOF/EC: RD TD > DCD (Red). TD = typically developing; ASD = autism spectrum disorder; DCD = developmental coordination; QA = quantitative anisotropy; MD = mean diffusivity; AD = axial diffusivity, and RD = radial diffusivity; CL = cingulum; Ca = caudal anterior; pfCL = parolfactory cingulum; bCC = body of the corpus callosum; FMi = forceps minor; ac = anterior commissure; MCP = middle cerebellar peduncle; pIFOF = posterior inferior fronto-occipital fasciculus; aIFOF = anterior inferior fronto-occipital fasciculus; EC = external capsule.

The whole brain correlational tractography analysis indicated group differences in all four diffusivities (QA, MD, AD, RD) in multiple tracts throughout the brain (Table 2; Fig. 1). Hyper- and hypo-diffusivity differences between ASD and other groups were found in the cingulum, subregions of the corpus callosum, middle cerebellar peduncle, inferior fronto-occipital fasciculus/external capsule (IFOF/EC), and other tracts (Table 2; Fig. 1). Group differences in diffusivity of u-fibers (ROI analysis) are reported in Table 3.

**Table 2:** Between group whole-brain diffusion differences and correlations with behavior in ASD and/or DCD.

Tracts	Group Contrast	Statistic	TD:ASD				TD: DCD				DCD:ASD			
			QA	MD	AD	RD	QA	MD	AD	RD	QA	MD	AD	RD
**Tracts unique to ASD and significant ASD group correlations (TD/DCD > ASD)**														
L parolfactory cingulum	QA: TD/DCD > ASD*	Tract #	95								87			
		*R*, *p*	**ASD**: AQC *R* = -.731 *p* = .002								**ASD**: AQC *R* = − .708 *p* = .003			
L frontal parietal cingulum	QA: TD/DCD > ASD*	Tract #	260		28						68		58	
		*R*, *p*	**ASD**: AQC *R* = − .736 *p* = .002		**ASD:** MABC-2 *R* = − .530 *p* = .023 ADI-R RSI *R* = − .488 *p* = .047						**ASD**: AQC *R* = − .742 *p* = .002		**ASD**: MABC-2 *R* = − .531 *p* = .023	
R frontal parietal cingulum	QA/AD: TD/DCD > ASD*	Tract #	518		116						280		21	
		*R*, *p*	**ASD**: AQC *R* = − .70 *p* = .004		**ASD:** AQC *R* = − .72 *p* = .002						**ASD**: AQC *R* = − .652 *p* = .008		**ASD**: AQC *R* = − .710 *p* = .003	
**Tracts where ASD is the extreme group compared to other groups (ASD > DCD > TD or TD > DCD > ASD) and significant ASD and DCD correlations**														
corpus callosum forceps minor/anterior commissure	QA: DCD > ASD > TD* MD: TD > DCD > ASD*	Tract #	44	266			24	1262			35	33		
		*R*, *p*	N/A	N/A			N/A	N/A			N/A	N/A		
L middle cerebellar peduncle	QA: ASD > DCD > TD*	Tract #	34				21				160			
		*R*, *p*	**ASD**: ADI-R RSI *R* = -.558 *p* = .020				N/A				**ASD**: ADI-R RSI *R* = − .54 *p* = .025 ADOS-2 *R* = − .44 *p* = .055 **DCD**: RBS *R* = − .53 *p* = .059			
**ASD vs TD differences and significant ASD correlations**														
R posterior IFOF/EC	QA: ASD > TD; AD: TD > ASD	Tract #	60		49									
		*R*, *p*	**ASD**: ADOS-2 *R* = − .465 *p* = .045		N/A									
corpus callosum genu	QA: TD > ASD	Tract #	140											
		*R*, *p*	**ASD**: AQC *R* = − .566 *p* = .028											
CC body	RD: TD > ASD† RD:ASD > DCD† MD:TD > ASD MD:ASD > DCD	Tract #		65		275						149		329
		*R*, *p*		**ASD:** CCR *R* = .441 *p* = .059		**ASD:** CCR *R* = .433 *p* = .064						N/A		**ASD**: CCR *R* = .455 *p* = .050

* = good-moderate overlap between group comparison tracts, † = moderate overlap between group comparison tracts;

*p* > .05; ASD = autism spectrum disorder; DCD = developmental coordination disorder; TD = typically developing; L = left; R = right; QA = quantitative anisotropy; MD = mean diffusivity; AD = axial diffusivity, and RD = radial diffusivity; AQC = Alexithymia Questionnaire for Children 2-factor Total ; SRS = Social Responsivity Scale-2 Total Score; RBS = Repetitive Behaviors Scale Total Score; SenSOR = Sensory Over-Responsivity Scale Total Score; MABC-2 = Movement Assessment Battery for Children-2 Total Score; CCR = Connors Child Report Total Score; GTC = gesture to command; IMI MF = imitation of meaningful gestures; IMI ML = imitation of meaningless gestures; TU = tool use; ADOS-2 = Autism Diagnostic Observational Scale-2 Comparison Score; ADI-R RSI = Autism Diagnostic Interview Revised, Reciprocal Social Interactions Scale.

**Table 3:** Significant DCD Specific Differences, TD > ASD/DCD Differences, and Between group differences.

Tracts	Group Contrast	Statistic	TD:ASD				TD: DCD				DCD:ASD			
			QA	MD	AD	RD	QA	MD	AD	RD	QA	MD	AD	RD
**Tracts unique to DCD (DCD > TD/ASD) and significant DCD group correlations**														
L cortico- descending	QA: DCD > TD DCD > ASD	Tract #					380				479			
		*R*, *p*					**DCD:** TU *R* = .545 *p* = .054				N/A			
**Tracts where DCD is the extreme group compared to other groups (TD > ASD > DCD) and significant ASD and DCD correlations**														
Bilateral CC forceps major/posterior thalamic radiation	QA: TD > ASD; TD > DCD* AD: TD > ASD > DCD*	Tract #	60		57		1239		343				122	
		*R*, *p*	N/A		N/A		**DCD:** RBS *R* = -.608 *p* = .027		N/A				**ASD**: AQC *R* = − .51 *p* = .048 MABC-2 *R* = .507 *p* = .032 **DCD:** N/A	
L CC forceps major/ posterior thalamic radiation	MD: TD > ASD > DCD*	Tract #		140				148				42		
		*R*, *p*		N/A				N/A				N/A		
**DCD vs TD differences and significant DCD correlations**														
R Anterior IFOF/EC	RD: TD > DCD	Tract #								147				
		*R*, *p*								**DCD:** MABC-2 *R* = -.642 *p* = .018				
R parahippo-campal cingulum	AD: TD > DCD	Tract #							81					
		*R*, *p*							**DCD:** SenSOR *R* = − .600 *p* = .030 RBS *R* = − .679 *p* = .0110					
Bilateral middle cerebellar peduncle	AD: TD > DCD	Tract #							108					
		*R*, *p*							N/A					
L cortico descending	QA: TD > DCD RD: DCD > TD	Tract #					380			125				
		*R*, *p*					N/A			N/A				
corpus callosum forceps minor	QA: TD > DCD	Tract #					24							
		*R*, *p*					N/A							
R cortico descending	QA: DCD > TD	Tract #					97							
		*R*, *p*					N/A							
R cortico-spinal tract	QA: DCD > TD	Tract #					97							
		*R*, *p*					N/A							
L cortico-spinal tract	AD: DCD > TD	Tract #							195					
		*R*, *p*							N/A					
L superior cortico-striatal tract	AD: DCD > TD	Tract #							184					
		*R*, *p*							N/A					
L cerebellum, internal tracts	QA: DCD > TD	Tract #					178							
		*R*, *p*					N/A							
**Tracts where both ASD and DCD differ compared to TD (TD > ASD/DCD) and significant ASD and DCD correlations**														
L parahippo-campal cingulum	AD: TD > ASDTD > DCD*	Tract #			41				70					
		*R*, *p*			N/A				**DCD:** TU *R* = − .566 *p* = .044					
corpus callosum forceps major/posterior thalamic radiation	RD: TD > ASDTD > DCD*	Tract #				95				88				
		*R*, *p*				N/A				N/A				
corpus callosum forceps minor	RD: TD > ASD TD > DCD*	Tract #				95				88				
		*R*, *p*				**ASD:** ADOS-2 *R* = − .44 *p* = .059				N/A				
R Posterior IFOF/EC	MD: TD > ASD TD > DCD*	Tract #		93				33						
		*R*, *p*		N/A				**DCD:** SenSOR *R* = − .569 *p* = .043						
L Posterior IFOF/EC	MD:TD > ASD TD > DCD† AD: TD > ASD	Tract #		325	67			105	36					
		*R*, *p*		N/A	N/A			**DCD:** TU *R* = − .568 *p* = .043	N/A					
Bilateral superior cerebellar peduncle	AD: TD > ASD TD > DCD†	Tract #			69				62					
		*R*, *p*			**ASD:** TU *R* = .467 *p* = .051				N/A					
**Tracts where ASD and DCD differentially differ from TD and significant ASD and DCD correlations**														
L cortico-spinal tract	QA: DCD > TD; TD > ASD†	Tract #	68				332							
		*R*, *p*	**ASD:** AQC *R* = − .56 *p* = .029				**DCD:** TU *R* = .611 *p* = .026							
R cerebellum, internal tracts	QA: DCD > TD TD > ASD ASD > DCD*	Tract #									2618			
		*R*, *p*	**ASD:** ADI-R *R* = − .45 *p* = .068				N/A				**ASD:** ADI-R *R* = − .487 *p* = .048 IRI *R* = .510 *p* = .026 **DCD:** N/A			

* = good-moderate overlap between group comparison tracts, † = moderate overlap between group comparison tracts;

cortico-descending consists of both cortico-spinal and cortico-pontine tracts. *p* > .05; ASD = autism spectrum disorder; DCD = developmental coordination disorder; TD = typically developing; L = left; R = right; QA = quantitative anisotropy; MD = mean diffusivity; AD = axial diffusivity, and RD = radial diffusivity; AQC = Alexithymia Questionnaire for Children 2-factor Total; SRS = Social Responsivity Scale-2 Total Score; RBS Total = Repetitive Behaviors Scale Total Score; SenSOR = Sensory Over-Responsivity Scale Total Score; MABC-2 Total = Movement Assessment Battery for Children-2 Total Score; CCR = Connors Child Report Total Score; GTC = gesture to command; IMI MF = imitation of meaningful gestures; IMI ML = imitation of meaningless gestures; TU = tool use; ADOS-2 = Autism Diagnostic Observational Scale-2 Comparison Score; ADI-R RSI = Autism Diagnostic Interview Revised, Reciprocal Social Interactions scale.

### Behavioral correlations

**Figure 2:**
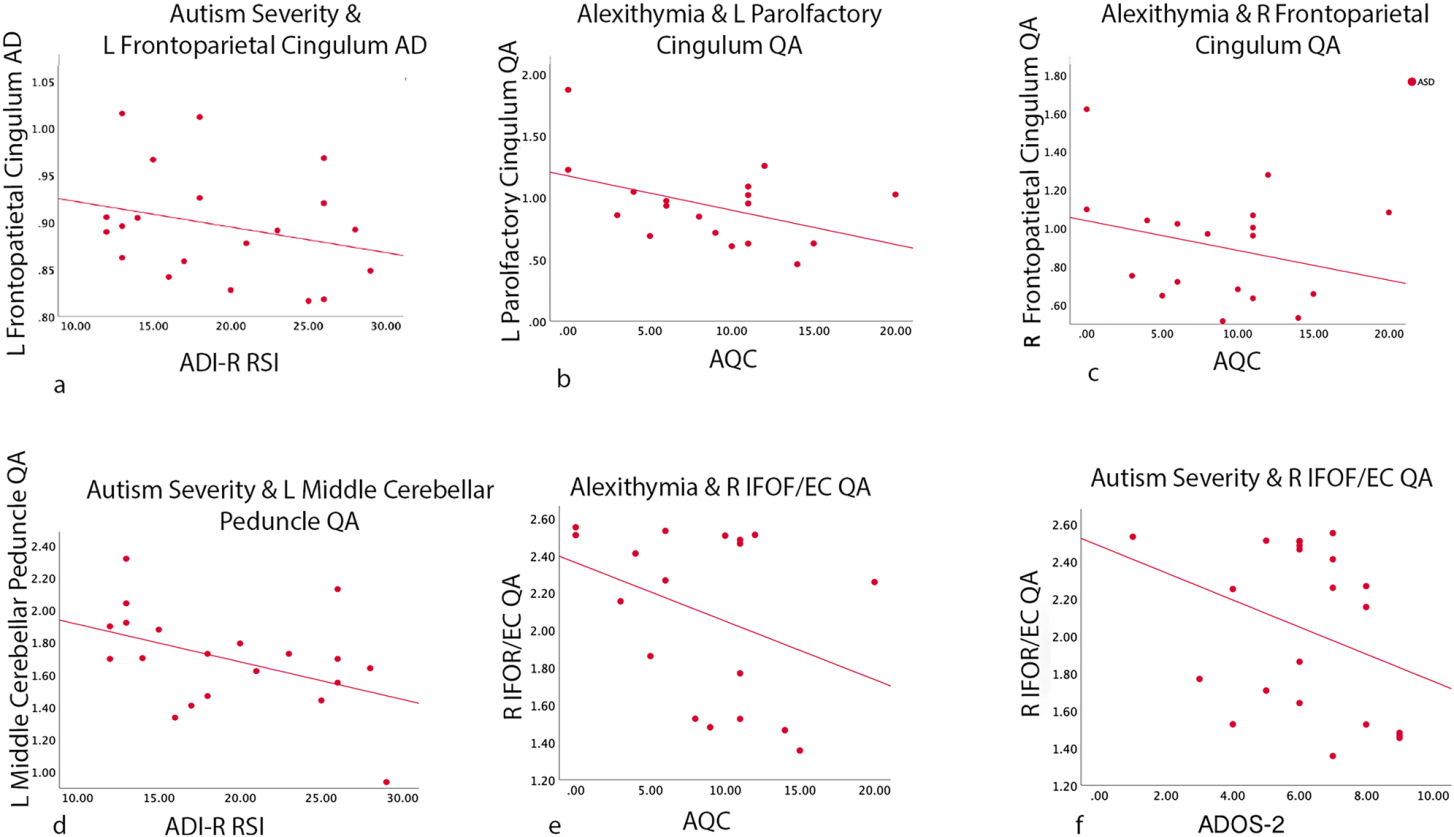
Relationship between diffusivity differences and autism severity or alexithymia. All correlations are within the ASD group and for TD > ASD contrasts. (**A**) Significant correlation between the left frontoparietal cingulum AD (TD > ASD contrast) and ADIR-RSI.; (**B**) Significant correlation between left parolfactory cingulum QA (TD > ASD contrast) and alexithymia (ACQ). (**C**) Significant correlation between right frontoparietal cingulum QA (TD > ASD contrast) and alexithymia. (**D**) Significant correlation between left middle cerebellar peduncle QA (TD > ASD contrast) and ADIR-RSI. (**E**) Significant correlation between right IFOF/EC QA (ASD > TD contrast) and alexithymia. (**F**) Significant correlation between right IFOF/EC QA (ASD > TD contrast) and ADOS-2 (autism severity). L = left; R = right; AQC = Autism Questionnaire for Children; ADIR-R RSI = Autism Diagnostic Interview Revised-Reciprocal Social Interactions; IFOF = inferior fronto-occipital fasciculus; EC = external commissure; TD = typically developing; ASD = autism spectrum disorder; DCD = developmental coordination disorder; QA = quantitative anisotropy; MD = mean diffusivity; AD = axial diffusivity, and RD = radial diffusivity.

Significant relationships between diffusivity and behavioral measures are reported in Tables 2, 3 and Fig. 2, as well as in Table 4 for cingulum u-fibers.

**Table 4:** U-fiber ROIs: Between group diffusion differences and significant ASD and DCD group correlations.

Tracts	Group Contrast	Statistic	TD:ASD				TD: DCD				DCD:ASD			
			QA	MD	AD	RD	QA	MD	AD	RD	QA	MD	AD	RD
Right Caudal anterior Ufiber	QA: DCD > TD > ASD AD: DCD > ASD	Tract #	652								652		652	
		Mean [SD] ***p*-value**	**TD:** 0.92 [0.17] **ASD:** 0.79 [0.19] **.0180**								**ASD**: 0.79 [0.19] **DCD**: 0.95 [0.12] **.00800**		**ASD**: 0.83 [0.022] **DCD**: 0.84 [0.017] **.0200**	
		*R*, *p*	**ASD:** AQC *R* = -.631 *p* = .0120								**ASD**: AQC *R* = -.631 *p* = .0120 **DCD:** N/A		**ASD**: AQC *R* = -.558 *p* = .031 **DCD**: SenSOR *R* = -.754 *p* = .0030	
Left Caudal Anterior U-fiber	DCD > ASD	Tract #	568								568		568	
		Mean [SD] ***p*-value**									**ASD**: 0.85 [0.22] **DCD**: 1.01 [0.15] **.0160**		**ASD**: 0.84 [0.024] **DCD:** 0.86 [0.022] **.0160**	
		*R*, *p*									**ASD**: AQC *R* = -.699 *p* = .00400 **DCD**: TU *R* = .627 *p* = .022		**ASD**: AQC *R* = -.672 *p* = .006 **DCD**: N/A	
Left Rostral Anterior U-fiber	DCD > ASD	Tract #									327			
		Mean [SD] ***p*-value**									**ASD**: 0.57 [0.14] **DCD**: 0.66 [0.11] **.0160**			
		*R*, *p*									N/A			
Left Posterior U-fiber	DCD > ASD	Tract #										603	603	
		Mean [SD] ***p*-value**										**ASD**: 0.59 [0.018] **DCD**: 0.60 [0.016] **.0190**	**ASD**: 0.83 [0.024] **DCD**: 0.84 [0.016] **.0280**	
		*R*, *p*										**ASD**: CCR *R* = .463 *p* = .046 **DCD**: N/A	**ASD**: AQC *R* = -.641 *p* = .010 SenSOR *R* = -.504 *p* = .0280 **DCD**: N/A	

ASD = autism spectrum disorder; DCD = developmental coordination disorder; TD = typically developing; L = left; QA = quantitative anisotropy; MD = mean diffusivity; AD = axial diffusivity, and RD = radial diffusivity; CC = corpus callosum; R = right; AQC = Alexithymia Questionnaire for Children 2-factor Total; SenSOR Total = Sensory Over-Responsivity Scale Total; MABC-2 Total = Movement Assessment Battery for Children-2 Total Score; CCR = Connor’s Child Report Total Score; TU = tool use.

## Discussion

Our behavioral results indicate that 80% of the ASD group have motor impairments as measured by the MABC-2, which are consistent with prior data on motor impairment in ASD^38–40^. Please refer to recent papers by our group in largely the same sample, which discuss in detail the behavioral differences between these two groups^18,41^. Here, we aimed to find the presence of specific white matter diffusivity differences in ASD that do not overlap with other developmental motor deficits, namely DCD. Thus, we focus on differences between ASD compared to the other groups and how those diffusivity differences correlate with behavioral measures. Finally, we consider tracts that are related to DCD.

### Specific patterns in the ASD group compared to the other two groups

Our data indicate that the most consistent structural “signatures” of ASD are the long-range fibers of the bilateral fronto-parietal cingulum and the left parolfactory cingulum (TD/DCD > ASD), as well as the right anterior caudal U-fibers (ASD > TD/DCD). While others have noted the potential importance of the cingulum in ASD^42^, this is the first study to identify the fronto-parietal and the parolfactory portions of the cingulum as well as the anterior caudal u-fibers as specific to core ASD symptomatology and not related to motor-related comorbidity. These results are discussed in detail below.

#### Long range cingulum fibers

The ASD group shows significantly reduced diffusivity compared to TD and DCD groups in the bilateral fronto-parietal cingulum (QA and AD) and the left parolfactory cingulum (ventral and anterior to fronto-parietal; QA). While these results are consistent with other studies indicating lower FA of the cingulum bundle is a common feature of ASD^6^, here we find *localized* differences in the fronto-parietal portion of the cingulum and compare them not only to a TD group but also to a DCD group. Thus, we can conclude that, in the left parolfactory cingulum and the right fronto-parietal cingulum, these ASD differences are not related to motor impairment comorbidity but related to core autism symptomatology. Accordingly, in the ASD group, we find that decreased QA and AD significantly correlate with greater severity of ASD symptomatology and greater alexithymia severity. These behavioral correlations suggest that autism severity and emotion deficits like alexithymia in ASD^43^ may be related to white matter abnormalities of the right frontoparietal and left parolfactory cingulum. By contrast, the left parolfactory cingulum correlates with both motor and emotional measures as well as autism severity, and thus ASD hypo-diffusivity here may be related to broader symptomatology.

The fronto-parietal cingulum connects the midcingulate (MCC) with many dorso-lateral prefrontal and dorsal motor regions. Consistent with its connectivity with motor regions, electrical stimulation in patients with epilepsy of this part of the cingulum bundle^22,44^, as well as of the MCC^9,45–47^, elicit complex motor behaviors often preceded by a desire to move (e.g., attempts to rise from the bed, eye saccades, and differential hand motor acts). Thus, this part of the cingulum and associated white matter bundles are crucial for orchestrating social behaviors, especially when triggered by strong motivations^9,46^.

The parolfactory sector of the cingulum connects the pregenual (pACC) and the subgenual anterior cingulate cortex (sACC) with many cortical and subcortical structures, such as the orbitofrontal cortex, ventrolateral prefrontal cortex, the anterior temporal pole, the ventral striatum/nucleus accumbens, and the lateral hypothalamus–all territories involved in emotional behaviors^9,48^. In line with its connectivity, electrical stimulation of the pACC produces interoceptive sensations and socio-emotional behaviors, in particular, laughter accompanied by mirth, as well as mirth induced by seeing another person laughing, imagining positive events, humor, and pleasant touch^45,48,49^. In contrast, intracortical recordings from the human sACC indicate strong firing rates when viewing negatively emotionally valanced stimuli^50^, in line with the well-known involvement of this cingulate sector in depression^3^. These data are in agreement with some studies showing that anterior and mid-cingulate cortices are also crucial for emotional awareness (the capacity for recognizing and understanding one's own emotions)^51^. Therefore, the reduced diffusivity of the bilateral fronto-parietal and of the left parolfactory cingulum in the ASD group compared to TD and DCD groups and their correlation with autism and alexithymia severity might be not only a structural “signature” of ASD, but also explain common ASD socio-emotional impairments, such as the difficulties in emotion processing, both for the self and others^52^.

#### Cingulum U-Fibers

In contrast to the reduced diffusivities in the fronto-parietal and parolfactory cingulum, the right caudal anterior cingulum u-fiber shows increased QA in ASD compared to both DCD and TD groups (though lower AD in the ASD group compared to the DCD group). Our results are in line with many electrophysiological studies showing that in ASD there is a reduction of the long-range functional connectivity^53–56^ and an increase in the local functional connectivity^57,58^. Reduced long-distance cortical–cortical reciprocal activity and coupling would impair the fundamental frontal function of integrating information from widespread and diverse systems (emotional, language, sensory, autonomic) and provide complex context-rich feedback, guidance, and control to lower-level systems. In addition, aberrantly heightened local frontal excitability in conjunction with impaired long-distance frontal cortical coupling with distant systems could explain frontal activity disturbances in ASD^58^. In fact, the stronger local connectivity between the anterior cingulate cortex and the adjacent medial prefrontal cortex could be the basis of an excess of prefrontal cortex activity in ASD, which may result in a lack of integration of emotional information conveyed by other cerebral territories. Our result that higher QA of the right caudal anterior cingulum u-fiber is correlated with alexithymia in the ASD group is in line with these notions.

### Tracts that show ASD as the extreme group (ASD > DCD > TD or TD > DCD > ASD)

Here we show that two tracts, the corpus callosum forceps minor/anterior commissure and the middle cerebral peduncle show diffusivity differences in the ASD group compared to both DCD and TD groups. Accordingly, these ASD differences are correlated with autism severity among other symptoms.

#### Corpus callosum forceps minor/anterior commissure

The forceps minor may connect prefrontal regions involved in social and cognitive functions^59^, and the anterior commissure (AC) connects the amygdalae and temporal lobes, involved in emotion, memory, and higher sensory processing^60,61^. As these two tracts are not easily distinguishable in our results, we discuss them together. In these tracts, the ASD group shows either significant hyper or hypo diffusivity compared to both groups (QA: ASD > DCD > TD; MD: TD > DCD > ASD; though note for RD: TD > ASD/DCD). In the ASD group, RD differences are nearly significantly related to greater autism severity (ADOS-2, *p* = 0.059). In relation to the TD vs. DCD findings, about 36% of individuals with DCD fall into the autism clinical range on social measures (SRS-2 SCI subscale)^12^. Thus, diffusivity differences in the forceps minor/AC may reflect social deficits that hallmark ASD and that are also sometimes found in DCD.

#### Left middle cerebellar peduncle

The middle cerebellar peduncle contains afferent fibers from the contralateral frontal, temporal, and occipital cortices and is the largest of three fiber tracts to the cerebellum. Here, we find that the left middle cerebellar peduncle shows significant differences in the ASD group compared to both groups (QA: ASD > DCD > TD). In addition, diffusivity differences in the ASD group are significantly correlated with autism severity. Our data are in line with a recent meta-analysis showing ASD diffusivity differences are common in the cerebellar peduncles^4^. In addition, dysfunction of the cerebellum, as well as congenital cerebellar hypoplasia/agenesis, have been associated with a number of autism-like behaviors^62,63^, such as poor eye contact and repetitive behaviors^64^. Interestingly, compared to TD, both ASD and DCD groups showed diffusivity differences in internal cerebellar connectivity as well as significantly lower AD in the superior cerebellar peduncle (a predominantly efferent pathway from deep cerebellar nuclei to the cortices, and strongly motoric in its function), consistently with prior studies^4,21,65^. Accordingly, diffusivity differences in the superior cerebellar peduncle (SCP) showed a trend with tool use (ASD group, AD: *p* = 0.051), supporting the notion that alterations of the SCP may be related to motor deficits rather than clinical group membership, in contrast to the decreased diffusivities of the middle cerebellar peduncle, which may be more related to autism core symptomatology.

### ASD vs. TD differences

#### Right posterior IFOF/extreme capsule

We were particularly interested in the IFOF/EC because, among other things, it connects temporal regions with inferior frontal gyrus (IFG). In ASD, compared to both TD and DCD groups, the IFG pars opercularis (IFGop) was previously found to be uniquely hypoactivate during observation of face and hand actions^12^. Accordingly, here we found that the bilateral posterior IFOF/EC shows less AD and MD in both ASD and DCD groups compared to the TD group, and these diffusivities in the left hemisphere are correlated with motor impairment (praxis skills of tool use—significant in DCD, and a trend in ASD [*p* = 0.071]). However, in the right posterior IFOF/EC, the ASD group alone additionally shows significantly higher QA and lower AD with respect to the TD group, which suggests additional abnormalities of the right posterior IFOF/EC in the ASD group compared to the other groups. In the ASD group, these diffusivity differences in the right posterior IFOF/EC significantly correlate with autism severity (QA: ADOS-2). These results are also in line with alterations in IFOF/EC diffusivity in ASD noted in previous studies^65–67^. Thus, while general IFOF/EC diffusivity differences may contribute to the motor symptomatology observed in both clinical groups, the additional diffusivity differences of the right posterior IFOF/EC, observed exclusively in the ASD group may be at the basis of specific ASD symptoms, such as social impairment, comprehension difficulties, and difficulty with facial expression and prosody during communicative behavior^25^. This latter interpretation is consistent with the notion that damage to the right posterior IFOF significantly affects the recognition of emotional facial expressions^68^ and with several pieces of evidence indicating that the posterior part of the right temporal and parietal lobes is crucial for the non-conscious perception of emotional faces as well as for prosody production and recognition^69^. Finally, the alteration of the IFOF/EC observed here could also contribute to the difficulties observed in ASD in language semantics and action understanding during social behavior, in line with the well-known roles of the posterior temporal cortex and the inferior frontal gyrus in these functions^70–72^.

#### Genu and body of corpus callosum

Our results indicate that the genu of the corpus callosum shows ASD vs. TD differences in QA and RD, and QA, and diffusivity significantly correlates with alexithymia in the ASD group. Since the genu of the corpus callosum laterally connects the left and right anterior cingulate cortices and lateral prefrontal^61,73^, our results are in line with the interhemispheric disconnectivity hypothesis of alexithymia. This hypothesis posits that poor interhemispheric connectivity between right hemisphere emotional processing regions and the left hemisphere, containing Broca’s area, results in the inability to verbalize emotions^74^.

The body of the corpus callosum also shows diffusivity differences between the ASD and the two other groups. In particular, we found lower MD in ASD compared to the TD group, but higher RD with respect to the DCD group, and these differences trend with ADHD symptomology. Further research is needed to determine if diffusivity differences in the body of the callosum are specific to ASD as other studies suggest^3,4^, or if they may reflect other forms of neurodevelopmental disorders^75^, including ADHD^76^.

### DCD related tracts

In general, we find that the DCD group has specific differences in motor-related tracts, and as one may expect, these correlate with motor and praxis skills. These results are briefly discussed below.

#### Tracts unique to DCD (DCD > TD/ASD)

Left Cortico-descending projections (cortico-spinal and cortico-pontine). We found significantly greater QA diffusivity in the left cortico-descending projections in the DCD group compared to both groups. As expected, diffusivity differences were correlated with motor ability (praxis skills of tool use). Thus, hypo-diffusivity in the cortico-spinal and cortico-pontine tracts may underlie DCD specific deficits.

#### Tracts where DCD is the extreme group (TD > ASD > DCD)

Corpus callosum (CC) forceps major/thalamic radiation. The forceps major is important for integrating visual, vestibular, somatosensory information for the guidance of body movements in space^72^ while the posterior thalamic radiation is involved in attention functions, such as the visual short-term memory capacity^73^. We found that in general, for the forceps major/thalamic radiation, the DCD group shows the lowest white matter diffusivity compared to TD and/or ASD (QA & AD: TD > ASD > DCD; though note for RD: TD > ASD/DCD). Our results are consistent with previous reports of DCD white matter diffusivity differences in the posterior CC (splenium)^12^ and with reports showing that individuals with DCD have poor visuo-motor and kinesthetic processing^74^. In the DCD group, we also find that decreased QA of the CC forceps major/thalamic radiation is significantly related to increased repetitive movements.

#### DCD vs. TD / Motor tracts and parahippocampal cingulum bundle

As you would expect, we find diffusivity differences in the DCD group compared to the TD group in several motor tracts, including the right cortico-spinal tract, the right cortico-descending tracts, the left superior corticostriatal tract, as well as the right parahippocampal cingulum (the left parahippocampal cingulum shows TD > ASD/DCD). For the left corticospinal tract, we find the two clinical groups show differential significant patterns from the TD group (QA: DCD > TD > ASD). Taken together, the DCD group shows diffusivity differences in a number of motor tracts compared to TD, and the left cortico-spinal tract in particular shows QA hyper-diffusivity in the DCD group (and hypo-diffivusivity in the ASD group compared to TD).

## General limitations

First, while here we tried to reduce variability by including only high functioning right-handed participants, future studies may consider increased within-group variability (left-handed, lower IQ, etc.) and larger sample sizes per group to better allow better generalizability of the results. Further, while we included children 8–17 and used age as a covariate in our analyses, white matter may develop at various rates in different groups^75^, and future studies may consider including age as a factor to better understand potential group differences through development. We note that all our participants are right-handed in order to control for potential laterality issues, and this should be kept in mind in generalizing the results. For example, the middle cingulate cortex, and thus likely its related white matter bundle, the cingulum, commonly shows contralateral motor representation^9^, and given the right handedness of our participants, generalizing laterality from our results should be proceeded with caution. In addition, many of our assessments were self-report (CCR, AQC) or parent report assessments (SenSOR, SRS-2), and future studies may further consider behavioral assessments.

## Conclusion

Here we show that the bilateral longitudinal fibers of the fronto-parietal cingulum and the right caudal anterior cingulum u-fibers show distinctive diffusivity differences in ASD. Although these tracts are involved in motor behavior, the comparison with DCD indicates that these diffusivity differences are not due to general developmental motor deficits but are potentially specific to ASD. Accordingly, we find correlations in these tracts with autism severity, and/or other symptomologies common to ASD. Further, we show that the corpus callosum forceps minor/anterior commissure and middle cerebellar peduncle show structural alterations that are the most significantly pronounced in the ASD group and are correlated with autism severity. Additionally, differences in QA and AD in the right posterior IFOF/EC are found in the ASD group compared to TD, and diffusivity in this tract is correlated with autism severity. This result may help clarify previous data indicating activation differences in ASD in cortical regions connected by this tract (IFGop)^12,42^ as well as socio-emotional processing differences in ASD. By contrast, DCD differences are most strongly found in the cortico-descending (cortico-spinal and cortico-pontine) tracts and other motor tracts. Future studies with increased sample sizes may help to elucidate whether such patterns between diffusivity differences and symptomatology can be discerned at the individual subject level.

## Supplementary Information

Supplementary Information.
