# Supplementary material for: Specific tractography differences in autism compared to developmental coordination disorder
Source: Sci Rep. 2022 Nov 14;12:19246. doi: 10.1038/s41598-022-21538-0 (PMC9663575; doi:10.1038/s41598-022-21538-0)
Supplement: Supplementary file 1 — Supplementary Information. [file 41598_2022_21538_MOESM1_ESM.docx]

# **Supplementary Materials**

1. **Supplementary Methods:**

Detailed Behavioral Measures: All data were recorded on RedCap.[^1,2^](https://www.zotero.org/google-docs/?BJJlKH)

Motor measures: *Motor performance skills:* The MABC-2[^3^](https://www.zotero.org/google-docs/?qREwh0) was used as a performance-based assessment that evaluates motor skill ability. The second (ages 7–10) and the third (ages 11–16) age bands were administered. The MABC-2 consists of three subtest scores: manual dexterity, gross-motor aiming and catching skills, and balance as well as a total score. Higher scores indicate better functioning. Item and subtest standard (scaled) total scores based on the normative sample were examined in our analyses. *Praxis skills*: Praxis performance was assessed using the Praxis Examination, a version of the Florida Apraxia Battery,[^4,5^](https://www.zotero.org/google-docs/?CiGqhw) modified for children.[^6^](https://www.zotero.org/google-docs/?S3UxKO) Children were asked to perform a variety of skilled gestures in three ways: by responding to 25 verbal commands (Gesture to Command, or GTC), 34 gestures performed by the examiner (Gesture to Imitation, or GTI), including 25 meaningful and 9 meaningless gestures (Meaningful Imitation (GTI MF) and Meaningless Imitation (GTI ML), and by demonstrating 17 gestures using an actual tool (Gesture with Tool Use, or GTU). The examination of each participant was video recorded from two perspectives and later scored independently by two occupational therapist raters. At least 80% concurrence between raters was achieved for each assessment to ensure the interrater reliability of scoring as outlined in Mostofky et al.[^6^](https://www.zotero.org/google-docs/?GsayrO) Each gesture was examined for the presence of errors according to criteria described in Mostofsky et al.[^6^](https://www.zotero.org/google-docs/?c3luRh) Total percent correct were the primary dependent measure of praxis performance. An item was scored as correct if no errors were made. Detailed descriptions of the praxis battery, scoring methodology, and reliability are provided in Mostofsky et al.[^6^](https://www.zotero.org/google-docs/?TPnmVq) and Dziuk et al.[^4^](https://www.zotero.org/google-docs/?dblBK5)

Social Measures. The Social Responsiveness Scale, 2nd Edition (SRS-2) is a parent-completed survey consisting of a total score, reflecting the severity of social deficits in ASD, and five subscales regarding their child’s social skill impairment: social awareness, social cognition, social communication, social motivation, and mannerisms. Scores are reported in T-scores.

Alexithymia measure. Alexithymia was measured using the 20-item self-report Alexithymia Questionnaire for Children (AQC),[^7^](https://www.zotero.org/google-docs/?QvcZuZ) an adapted version of the Toronto Alexithymia Scale. Three scores were used to assess alexithymia: difficulty identifying feelings (AQC ID), difficulty describing and communicating feelings (AQC COMM), and the total of these two factors, AQC two-factor total.[^8^](https://www.zotero.org/google-docs/?Zs7xxA)

Autism Severity Measures. The ADOS-2[^9^](https://www.zotero.org/google-docs/?POjyi3) is a semi-structured, standardized assessment of communication, social interaction, play, and imagination designed for use in diagnostic evaluations of individuals referred for a possible Autism Spectrum Disorder (ASD). Comparison scores range from 1 to 10. Higher scores reflect increased ASD characteristics. The ADI-R is a clinical diagnostic instrument for assessing autism. It is a structured interview with the parent with open-ended questions and scored across three domains: Language/Communication (LC), Reciprocal Social Interactions (RSI), and Restricted, Repetitive, and Stereotyped Behaviors (RRB). In this study, RSI was used as our index of autism social severity since we were primarily interested in social severity.

Measures of Sensory Responsivity. The Sensory Over-Responsivity (SenSOR) Inventory is a validated questionnaire that assesses a child’s sensory over-responsivity (completed by parents).[^10^](https://www.zotero.org/google-docs/?Et8ahB)

Repetitive behaviors: The Repetitive Behavior Scale-Revised (RBS-R) is a validated 43-item questionnaire that measures repetitive behaviors in children, adolescents, and adults with ASD, appropriate for ages 2-18 years.[^11,12^](https://www.zotero.org/google-docs/?KWugMX)

Attention Deficit Hyperactivity Disorder: The Conners 3^rd^ edition (Conners3) ADHD Index (Conners, C. K. Conners 3rd edition)[^13^](https://www.zotero.org/google-docs/?bnqr6k) for ages 6 through 18 years, consists of 10 items from the larger Conners scale that best differentiate children with and without ADHD. The child self-report measure was used.

Data collection. DWI was acquired on a three Tesla MAGNETOM Prisma (Siemens, Erlangen, Germany) using a 20-channel head coil and the following parameters: A multi-shell diffusion scheme was used, with b-values of approximately 1500 and 3000 s/mm2. The number of diffusion sampling directions were 90 collected in the AP and PA direction separately. The slice thickness was 1.5 mm, number of slices =92; Flip angle =78˚; matrix size = 140x140 ; FOV=210x210 mm^2^; TR=3222 ms; TE=89.2 ms; average = 1, Multiband factor = 4. A structural scan also was acquired for each participant (T1-weighted MPRAGE; TR = 1,950 ms, TE = 3.09 ms, flip angle = 10˚, 256 x 256 matrix, 208 coronal slices, 1 mm isotropic resolution). The total acquisition time was 14 minutes.

Motion Analysis. All participant’s data (N=85) was checked for excessive motion. Twenty-six participants were removed for excessive motion (TD=13 ASD=6 DCD=7; Figure S1). For the remaining 59 participants, the mean motion was less than 1mm. There were no between-group differences in motion in the final sample; the TD group had the most motion (mean 0.166, SD:0.100) followed by the ASD group (mean 0.172, SD: 0.0995) and then the DCD group (mean 0.131, SD: 0.148; Figure S1).


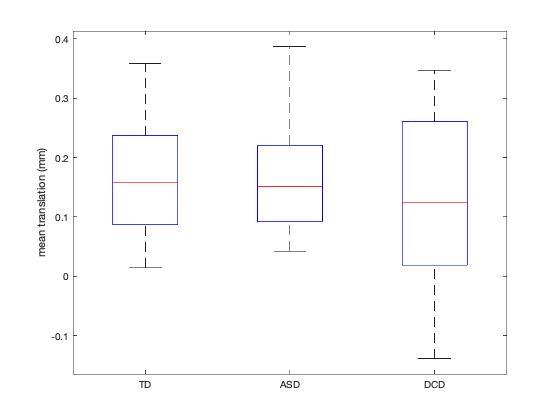


**Suppl. Figure 1: Box plot of translational motion parameters (mm) from each group.** TD=Typically developing; ASD=autism spectrum disorder; DCD=developmental coordination disorder.

Data preprocessing. Each participant’s diffusion-weighted image was processed using TORTOISE [^14,15^](https://www.zotero.org/google-docs/?ZQFa9J) to perform the motion, eddy current correction, and EPI distortion correction. BLip-correction was performed using DR-BUDDI [^16^](https://www.zotero.org/google-docs/?XHFdpu). Processed images were analyzed by DSI Studio. Q-space diffeomorphic reconstruction was then performed in HCP1021 template space, the quantitative anisotropy was extracted as the local connectome fingerprint and used in the connectometry analysis.[^17^](https://www.zotero.org/google-docs/?l5OvVw)

Correlational Analyses: To identify and segment tracts for correlational analysis from the whole brain group results, *DSI STUDIOS Recognize and Cluster* function was applied to whole brain group contrast tractography maps. Each individual track was checked to confirm its correctness to classic anatomical parcellations. In instances where multiple tracts were clustered inconsistently, tracts were separated by hand based on anatomy. Diffusivities (QA, MD, AD, RD) were then extracted via DSI STUDIOS. Tracts with less than 20 streamlines were considered too few to obtain reliable results and were subsequently discarded. Partial correlations were conducted on the remaining tracts controlling for age, sex, and full-scale IQ. Each significant correlation was plotted to correct for the presence of outliers.

Group contrast patterns across multiple contrasts:

Individual anatomical tracks with multiple significant group contrasts (e.g., TD>ASD and TD>DCD) were visually inspected for the degree of overlap between significant different contrasts. A good-moderate overlap rating indicates tracks overlapped more than 50%, a moderate overlap rating indicates tracts overlapped less than 50%. Tracks not indicated shared very little to no overlap.

Statistical Limitations.

When extracting diffusivity metrics from individual ROIs, reproduction of the whole brain level results was achieved for the majority of the parcellated ROI tracks. However, in 11 ROIs, we were unable to reproduce whole-brain results, as the whole brain analysis utilizes bootstrap-resampling to derive statistical tests and to minimize the effect of outliers, a method that does not apply to the ROI analysis. For this reason, means and SDs are not reported in the whole brain analysis, as they are not reflective of the algorithm used by that analysis. To minimize the effect of this methodological discrepancy, in our correlational analyses, all correlations were plotted to make sure they were not driven by outliers, and outliers were removed. Finally, while we aimed to investigate group differences between three groups, DSI Studios only allows for whole brain group contrast analysis to be performed between up to two groups. Thus, patterns found in the same region in all group contrasts (e.g., TD:ASD, TD:DCD, ASD:DCD) have varying degrees of streamline overlap. Thus, we indicate in our results the degrees of overlap in these group contrasts when they occur in the same region (see *Supplementary Materials* for more detail*)*. Interpretation of findings in these instances should be taken with caution and warrant further studies.

**Table 1S. Behavioral Group differences**

| Dependent  Variable | Group (A) | Group (B) | Mean  Difference (A-B) | Std.  Error | P-Value | 95% Confidence  Interval | | |
| --- | --- | --- | --- | --- | --- | --- | --- | --- |
| Lower Bound Upper Bound | | | | | | | |  |
| Sex | TD | ASD | 0.108 | 0.147 | 0.466 | -0.187 | 0.403 |  |
|  |  | DCD | 0.006 | 0.160 | 0.971 | -0.315 | 0.327 |  |
|  | ASD | TD | -0.108 | 0.147 | 0.466 | -0.403 | 0.187 |  |
|  |  | DCD | -0.102 | 0.159 | 0.522 | -0.420 | 0.216 |  |
|  | DCD | TD | -0.006 | 0.160 | 0.971 | -0.327 | 0.315 |  |
|  |  | ASD | 0.102 | 0.159 | 0.522 | -0.216 | 0.420 |  |
| Age | TD | ASD | 0.575 | 0.668 | 0.392 | -0.762 | 1.913 |  |
|  |  | DCD | 0.189 | 0.726 | 0.795 | -1.266 | 1.644 |  |
|  | ASD | TD | -0.575 | 0.668 | 0.392 | -1.913 | 0.762 |  |
|  |  | DCD | -0.386 | 0.719 | 0.593 | -1.827 | 1.054 |  |
|  | DCD | TD | -0.189 | 0.726 | 0.795 | -1.644 | 1.266 |  |
|  |  | ASD | 0.386 | 0.719 | 0.593 | -1.054 | 1.827 |  |
| Full Scale IQ | TD | ASD | -2.393 | 4.960 | 0.631 | -12.329 | 7.541 |  |
|  |  | DCD | 1.083 | 5.395 | 0.842 | -9.724 | 11.890 |  |
|  | ASD | TD | 2.393 | 4.960 | 0.631 | -7.541 | 12.329 |  |
|  |  | DCD | 3.477 | 5.341 | 0.518 | -7.223 | 14.177 |  |
|  | DCD | TD | -1.083 | 5.394 | 0.842 | -11.890 | 9.724 |  |
|  |  | ASD | -3.477 | 5.341 | 0.518 | -14.177 | 7.223 |  |
| AQC | TD | ASD | -1.857 | 1.5021 | 0.222 | -4.871 | 1.157 |  |
|  |  | DCD | -0.690 | 1.552 | 0.658 | -3.805 | 2.424 |  |
|  | ASD | TD | 1.857 | 1.502 | 0.222 | -1.157 | 4.871 |  |
|  |  | DCD | 1.167 | 1.607 | 0.471 | -2.058 | 4.391 |  |
|  | DCD | TD | 0.690 | 1.552 | 0.658 | -2.424 | 3.805 |  |
|  |  | ASD | -1.167 | 1.607 | 0.471 | -4.391 | 2.058 |  |
| CCR | TD | ASD | -5.368* | 1.915 | 0.007 | -9.203 | -1.533 |  |
|  |  | DCD | -4.408* | 2.083 | 0.039 | -8.580 | -0.236 |  |
|  | ASD | TD | 5.368* | 1.915 | 0.007 | 1.533 | 9.203 |  |
|  |  | DCD | 0.960 | 2.062 | 0.643 | -3.171 | 5.091 |  |
|  | DCD | TD | 4.408* | 2.083 | 0.039 | 0.236 | 8.580 |  |
|  |  | ASD | -0.960 | 2.062 | 0.643 | -5.091 | 3.171 |  |
| SRS Total | TD | ASD | -32.667* | 2.178 | <.001 | -37.031 | -28.303 |  |
|  |  | DCD | -9.860* | 2.342 | <.001 | -14.553 | -5.168 |  |
|  | ASD | TD | 32.667* | 2.178 | <.001 | 28.303 | 37.03 |  |
|  |  | DCD | 22.807* | 2.342 | <.001 | 18.114 | 27.500 |  |
|  | DCD | TD | 9.860* | 2.342 | <.001 | 5.168 | 14.553 |  |
|  |  | ASD | -22.807* | 2.3415 | <.001 | -27.499 | -18.114 |  |
| MABC-2 Total | TD | ASD | 4.905* | 0.616 | <.001 | 3.670 | 6.140 |  |
|  |  | DCD | 6.259* | 0.662 | <.001 | 4.931 | 7.587 |  |
|  | ASD | TD | -4.905* | 0.616 | <.001 | -6.140 | -3.67 |  |
|  |  | DCD | 1.354* | 0.662 | 0.046 | 0.0264 | 2.682 |  |
|  | DCD | TD | -6.259* | 0.663 | <.001 | -7.587 | -4.931 |  |
|  |  | ASD | -1.354* | 0.663 | 0.046 | -2.682 | -0.026 |  |
| RBS Total | TD | ASD | -22.107* | 3.533 | <.001 | -29.191 | -15.023 |  |
|  |  | DCD | -3.563 | 3.793 | 0.352 | -11.167 | 4.042 |  |
|  | ASD | TD | 22.107* | 3.533 | <.001 | 15.023 | 29.191 |  |
|  |  | DCD | 18.544* | 3.753 | <.001 | 11.021 | 26.068 |  |
|  | DCD | TD | 3.563 | 3.793 | 0.352 | -4.042 | 11.167 |  |
|  |  | ASD | -18.545* | 3.753 | <.001 | -26.068 | -11.021 |  |
| SenSOR Total | TD | ASD | -20.833* | 2.899 | <.001 | -26.640 | -15.026 |  |
|  |  | DCD | -6.771* | 3.153 | 0.036 | -13.087 | -0.454 |  |
|  | ASD | TD | 20.833* | 2.899 | <.001 | 15.026 | 26.640 |  |
|  |  | DCD | 14.063* | 3.122 | <.001 | 7.808 | 20.317 |  |
|  | DCD | TD | 6.771* | 3.153 | 0.036 | 0.454 | 13.087 |  |
|  |  | ASD | -14.063* | 3.122 | <.001 | -20.317 | -7.808 |  |
| GTC | TD | ASD | .160* | 0.047 | 0.001 | 0.065 | 0.254 |  |
|  |  | DCD | .103* | 0.051 | 0.048 | 0.001 | 0.204 |  |
|  | ASD | TD | -.160* | 0.047 | 0.001 | -0.254 | -0.065 |  |
|  |  | DCD | -0.060 | 0.051 | 0.267 | -0.159 | 0.045 |  |
|  | DCD | TD | -.103* | 0.051 | 0.048 | -0.204 | -0.001 |  |
|  |  | ASD | 0.057 | 0.051 | 0.267 | -0.045 | 0.159 |  |
| IMI MF | TD | ASD | .298* | 0.049 | <.001 | 0.200 | 0.396 |  |
|  |  | DCD | .192* | 0.053 | <.001 | 0.087 | 0.297 |  |
|  | ASD | TD | -.298* | 0.049 | <.001 | -0.400 | -0.200 |  |
|  |  | DCD | -.106* | 0.053 | 0.049 | -0.211 | -0.000 |  |
|  | DCD | TD | -.192* | 0.053 | <.001 | -0.297 | -0.087 |  |
|  |  | ASD | .106* | 0.053 | 0.049 | 0.0003 | 0.211 |  |
| IMI ML | TD | ASD | .184* | 0.053 | <.001 | 0.078 | 0.289 |  |
|  |  | DCD | .216* | 0.057 | <.001 | 0.102 | 0.300 |  |
|  | ASD | TD | -.184* | 0.053 | <.001 | -0.289 | -0.078 |  |
|  |  | DCD | 0.032 | 0.057 | 0.575 | -0.082 | 0.145 |  |
|  | DCD | TD | -.216* | 0.057 | <.001 | -0.329 | -0.102 |  |
|  |  | ASD | -0.032 | 0.057 | 0.575 | -0.145 | 0.082 |  |
| TU | TD | ASD | .264* | 0.045 | <.001 | 0.175 | 0.354 |  |
|  |  | DCD | .218* | 0.048 | <.001 | 0.122 | 0.315 |  |
|  | ASD | TD | -.264* | 0.045 | <.001 | -0.354 | -0.175 |  |
|  |  | DCD | -0.046 | 0.048 | 0.342 | -0.143 | 0.050 |  |
|  | DCD | TD | -.218* | 0.048 | <.001 | -0.315 | -0.121 |  |
|  |  | ASD | 0.046 | 0.048 | 0.342 | -0.050 | 0.143 |  |

* *p*<.05

AQC=Alexithymia Questionnaire for Children 2-factor Total; SRS Total=Social Responsivity Scale Total; RBS Total=Repetitive Behaviors Scale Total; SenSOR Total=Sensory Over-Responsivity Scale Total; MABC-2 Total=Movement Assessment Battery for Children-2 Total Score; CCR= Connors Child Report; GTC=gesture to command; IMI MF=imitation of meaningful gestures; IMI ML=imitation of meaningless gestures; TU=tool use

**Table 2S. Significant whole-brain group differences in QA, MD, AD, and RD: ASD compared to DCD only.**

| Group Contrast |  |  | TD:ASD | | | | TD: DCD | | | | DCD:ASD | | | |
| --- | --- | --- | --- | --- | --- | --- | --- | --- | --- | --- | --- | --- | --- | --- |
|  | Group Contrast | Track # |  | | | |  | | | |  | | | |
| Tracts |  |  | QA | MD | AD | RD | QA | MD | AD | RD | QA | MD | AD | RD |
| **ASD vs DCD differences** | | | | | | | | | | | | | | |
| L anterior cortico-striatal tract | DCD>ASD | Tract # |  |  |  |  |  |  |  |  |  | 31 |  |  |
| Bilateral middle cerebellar peduncle | DCD>ASD | Tract # |  |  |  |  |  |  |  |  |  |  | 40 |  |
| Left SLF1 | DCD>ASD | Tract # |  |  |  |  |  |  |  |  |  |  | 31 |  |
| L anterior thalamic radiation | DCD>ASD | Tract # |  |  |  |  |  |  |  |  |  | 43 |  |  |
| R parietal aslant tract | ASD>DCD | Tract # |  |  |  |  |  |  |  |  |  | 312 |  |  |
| R posterior thalamic radiation/CC forceps major | ASD>DCD | Tract # |  |  |  |  |  |  |  |  |  | 53 |  |  |
| L anterior cortico-striatal tract | ASD>DCD | Tract # |  |  |  |  |  |  |  |  |  | 31 |  |  |
| L cerebellum, internal tracts | ASD>DCD | Tract # |  |  |  |  |  |  |  |  | 460 |  |  |  |
| CC body | ASD>DCD | Tract # |  |  |  |  |  |  |  |  |  | 149 |  | 329 |

SLF=superior longitudinal fasciculus; L=left; R=right; ASD=autism spectrum disorder; DCD=developmental coordination disorder; TD=typically developing.

**Supplemental References**

[1. Harris PA, Taylor R, Thielke R, Payne J, Gonzalez N, Conde JG. Research electronic data capture (REDCap)--a metadata-driven methodology and workflow process for providing translational research informatics support. *J Biomed Inform*. 2009;42(2):377-381. doi:10.1016/j.jbi.2008.08.010](https://www.zotero.org/google-docs/?LuljVQ)

[2. Harris PA, Taylor R, Minor BL, et al. The REDCap consortium: Building an international community of software platform partners. *J Biomed Inform*. 2019;95:103208. doi:10.1016/j.jbi.2019.103208](https://www.zotero.org/google-docs/?LuljVQ)

[3. Henderson SE, Sugden DA, Barnett AL. Movement Assessment Battery for Children-2 - PsycNET. Published 2007. Accessed November 24, 2021. https://doi.apa.org/doiLanding?doi=10.1037%2Ft55281-000](https://www.zotero.org/google-docs/?LuljVQ)

[4. Dziuk MA, Larson JCG, Apostu A, Mahone EM, Denckla MB, Mostofsky SH. Dyspraxia in autism: association with motor, social, and communicative deficits. *Dev Med Child Neurol*. 2007;49(10):734-739. doi:10.1111/j.1469-8749.2007.00734.x](https://www.zotero.org/google-docs/?LuljVQ)

[5. Rothi G, Raymer A, Ochipa C, Maher L, Greenwald M, Heilman K. *Florida Apraxia Battery-Revised*.; 2003.](https://www.zotero.org/google-docs/?LuljVQ)

[6. Mostofsky SH, Dubey P, Jerath VK, Jansiewicz EM, Goldberg MC, Denckla MB. Developmental dyspraxia is not limited to imitation in children with autism spectrum disorders. *J Int Neuropsychol Soc*. 2006;12(03). doi:10.1017/S1355617706060437](https://www.zotero.org/google-docs/?LuljVQ)

[7. Rieffe C, Oosterveld P, Terwogt M. An alexithymia questionnaire for children: Factorial and concurrent validation results. *Personal Individ Differ*. 2006;40:123-133. doi:10.1016/j.paid.2005.05.013](https://www.zotero.org/google-docs/?LuljVQ)

[8. Loas G, Braun S, Delhaye M, Linkowski P. The measurement of alexithymia in children and adolescents: Psychometric properties of the Alexithymia Questionnaire for Children and the twenty-item Toronto Alexithymia Scale in different non-clinical and clinical samples of children and adolescents. *PLOS ONE*. 2017;12(5):e0177982. doi:10.1371/journal.pone.0177982](https://www.zotero.org/google-docs/?LuljVQ)

[9. Lord C, Rutter M, DiLavore PC, Risi S, Gotham K, Bishop S. *Autism Diagnostic Observation Schedule, Second Edition (ADOS-2)*. Western Psychological Services; 2012.](https://www.zotero.org/google-docs/?LuljVQ)

[10. Schoen SA, Miller LJ, Green KE. Pilot Study of the Sensory Over-Responsivity Scales: Assessment and Inventory. *Am J Occup Ther*. 2008;62(4):393-406. doi:10.5014/ajot.62.4.393](https://www.zotero.org/google-docs/?LuljVQ)

[11. Hooker JL, Dow D, Morgan L, Schatschneider C, Wetherby AM. Psychometric analysis of the repetitive behavior scale-revised using confirmatory factor analysis in children with autism. *Autism Res*. 2019;12(9):1399-1410. doi:10.1002/aur.2159](https://www.zotero.org/google-docs/?LuljVQ)

[12. Schertz HH, Odom SL, Baggett KM, Sideris JH. Parent-Reported Repetitive Behavior in Toddlers on the Autism Spectrum. *J Autism Dev Disord*. 2016;46(10):3308-3316. doi:10.1007/s10803-016-2870-x](https://www.zotero.org/google-docs/?LuljVQ)

[13. Conners CK. Conners 3rd Edition. multi-health-systems-usd. Published 2008. Accessed November 30, 2021. https://storefront.mhs.com/collections/conners-3](https://www.zotero.org/google-docs/?LuljVQ)

[14. Irfanoglu MO, Nayak A, Jenkins J, Pierpaoli C. TORTOISE v3: Improvements and New Features of the NIH Diffusion MRI Processing Pipeline. *Int Soc Magn Reson Med*. Published online 2017. https://archive.ismrm.org/2017/3540.html](https://www.zotero.org/google-docs/?LuljVQ)

[15. Pierpaoli C, Walker L, Irfanoglu MO, et al. TORTOISE: An Integrated Software Package for Processing of Diffusion MRI Data. *Int Soc Magn Reson Med*. Published online 2010. https://archive.ismrm.org/2010/1597.html](https://www.zotero.org/google-docs/?LuljVQ)

[16. Irfanoglu MO, Modi P, Nayak A, Hutchinson EB, Sarlls J, Pierpaoli C. DR-BUDDI (Diffeomorphic Registration for Blip-Up blip-Down Diffusion Imaging) Method for Correcting Echo Planar Imaging Distortions. *NeuroImage*. 2015;106:284-299. doi:10.1016/j.neuroimage.2014.11.042](https://www.zotero.org/google-docs/?LuljVQ)

[17. Yeh FC, Badre D, Verstynen T. Connectometry: A statistical approach harnessing the analytical potential of the local connectome. *NeuroImage*. 2016;125:162-171. doi:10.1016/j.neuroimage.2015.10.053](https://www.zotero.org/google-docs/?LuljVQ)
